# Sustainable Growth of Social Tourism: A Growth Mixture Modeling Approach Using Heterogeneous Travel Frequency Trajectories

**DOI:** 10.3390/ijerph18105241

**Published:** 2021-05-14

**Authors:** Jaeseok Lee, Jooa Baek

**Affiliations:** 1 Faculty of Hospitality and Tourism Management, Macau University of Science and Technology, Macao, China; jaeseoklee@must.edu.mo; 2 Doctor of Business Administration Program, Goldey-Beacom College, Wilmington, DE 19808, USA

**Keywords:** travel frequency trajectory, growth mixture modeling, latent growth curve modeling, longitudinal data analysis, sustainable social tourism

## Abstract

As travel activity has gained attention as one of the essential ways of understanding the sustainable growth of social tourism, a growing number of research projects have been conducted to elucidate the relationship between residents’ travel quantity (frequency) and quality (experience) in both macro and micro perspectives. Yet, very little research has highlighted that travel opportunities are not equally available to residents, especially a longitudinal perspective. The current study classified domestic travelers into four distinct classes using four years of longitudinal data from 5054 Korean residents. Latent growth curve modeling (LGCM) and growth mixture modeling (GMM) were employed to find out (1) the optimal number of classes, (2) the longitudinal travel frequency trajectory of each class, and (3) the distinctive demographic and travel characteristics of the four classes. This study provides some practical implications for policymakers when optimizing available resources for sustainable travel opportunities to relevant target sub-populations. Furthermore, detailed step-by-step analytic tutorials are also introduced for the extended application of longitudinal latent variable analysis in the tourism and hospitality fields, providing additional insights for relevant stakeholders.

## 1. Introduction

Tourism experience enriches individuals and society as a whole. Chen and Petrick [1] summarized the health and wellness benefits of travel experiences from previous studies and concluded that positive tourism experiences make travelers feel relaxed, healthier, less stressful, and, eventually, happier. Similarly, McCabe and Qiao [2] assert that tourism should be considered from a welfare perspective due to its positive effects on sustainability, mental health, quality of life, responsible tourism, and care-giving. Thus, these positive effects contribute to overall well-being and life satisfaction in general [3,4]. Social tourism, a form of social welfare provided by the government, is a clear example of a related tourism policy. Social tourism enhances the quality of life of all residents by providing additional opportunities to enjoy travel experiences, including those with a limited ability (e.g., money, time, and health) [5,6,7,8,9,10]. The World Tourism Organization adopted the Global Code of Ethics for Tourism in 1999, where the right to tourism is noted to address the importance of social tourism [11]. Similarly, the International Social Tourism Organisation emphasizes the importance of “Tourism for all” in an attempt to expand social tourism across the continents [8]. Alvarez-Sousa [10] and Griffin and Stacey [9] echoed the importance of a tourism-for-all policy in Europe and asserted that more research should be conducted globally.

Accordingly, many governments have put considerable effort into promoting leisure, recreation, and tourism activities. The increasing public consensus on social tourism started in the 1970s in South Korea, resulting in the establishment of the Tourism Basic Act in 1975 and the long-term Korean tourism development and promotion plan in 1979 [12]. Since then, the Korean government has developed various tourism-related laws, projects, and policies that aim to promote tourism activities for domestic tourists (e.g., Travel Week and TourDure project) [13]. More recently, private sectors have been actively engaged in social tourism offerings (e.g., cultural, recreational, and art activities) as part of their corporate social responsibility [14]. Although various efforts made by the Korean government and private sectors have ultimately promoted the growth of domestic tourism and the local economies of tourist destinations, the unique collaboration between public and private sectors in social tourism raises the question of whether tourism-for-all is implemented in Korea as it intended.

Meanwhile, the majority of social tourism studies have focused largely on cross-sectional outcomes, such as the overall impact on national tourism at a given time [10,15]. To the best of our knowledge, the year-on-year trends in travel patterns in terms of the individual-level travel frequency have been neglected in hospitality and tourism journals. Therefore, a longitudinal analysis at the individual level may provide additional insights into the tourism policy and its effect on individual behaviors. For example, the individual-level travel frequency pattern and their length of stay could help policymakers determine tourism demand at the national level [16,17], which eventually connects to nationwide tourism policy and/or strategy. More importantly, the understanding of travel frequency at the individual level and its determining factors are directly linked to the fluctuation and profitability of the domestic tourism market.

Recently, Xu and Martinez [18] called for further research based on LGCM to analyze repeated measures of longitudinal data in the hospitality and tourism fields. Besides, researchers usually adopt a family of longitudinal analysis (e.g., repeated measures analysis of variance, latent curve model, and panel data analysis) as their research design when exploring the changes in the outcome variable over time. These methods allow scholars to understand the overall trend over time based on the assumption of a homogenous population. Nevertheless, numerous hospitality and tourism studies have exclusively utilized cross-sectional research design to understand complex social phenomena and possibly neglect within-subject variations over time [18,19].

Furthermore, research recently published in hospitality and tourism fields strongly emphasized the importance of heterogeneity within the sample to get a clearer understanding of research questions [20,21]. To respond to these calls, researchers may need to incorporate person-centered (differences among unobserved subpopulations) and variable-centered (differences in outcome change) approaches into the analytic framework. Therefore, we suggest growth mixture modeling (GMM) as one of the possible analytic tools for solving the research question. GMM is “a method for identifying multiple unobserved sub-populations, describing longitudinal change within each unobserved sub-population, and examining differences in change among unobserved sub-populations” [22] (p. 565). In contrast to traditional longitudinal analytic techniques, GMM, as part of the family of latent variable modeling techniques, can identify unobserved heterogeneous subgroups among the population, thereby helping researchers understand heterogeneity in trajectories over time [23]. Notably, segmentation with cross-sectional data ignores the nonindependence of within-subject variations and the dynamic nature of between-subject variations over time [19]. However, GMM enables researchers to identify different sets of heterogeneous subpopulations (often called latent classes), where homogeneous trajectories (i.e., intercept and slope factors) are shared by within-class individuals. In other words, GMM can simultaneously cluster and describe changes in the outcome variable over time. Furthermore, it flexibly adds the predictors and distal outcomes to the trajectory, as well as the class membership variable. In a similar way to multinomial logistic and ordinary regression analysis, GMM analyzes the relationship between predictors and latent class membership, and between the membership and distal outcomes depending on the scale of the distal outcome variable(s).

Therefore, the current study aims (**1**) to examine the sustainable growth of social tourism in each heterogeneous social stratum with a focus on the Korean domestic travel market from a longitudinal perspective, and (**2**) to illustrate a step-by-step guide on the application of GMM. To do so, this study first explains the basic foundation of the GMM analytic framework in easy-to-understand language to facilitate the use of the statistical method in future studies for researchers with minimal statistical knowledge. This study uses nationwide panel survey data to illustrate the analytic procedure and the interpretation of the findings. Using the South Korean domestic travel frequency data, the present study identifies the trajectories of travel frequency among Korean tourists over time and further investigates the differences in demographic characteristics across distinct groups. In addition, we recommend further research topics that can utilize GMM in the hospitality and tourism fields.

## 2. Literature Review

### 2.1. Social Tourism

The history of social tourism can be traced back to the nineteenth century, mostly in Europe, and the definition has changed throughout global historic events such as World War II and The Great Depression [2]. The recent definition of social tourism, “all activities, relationships and phenomena in the field of tourism resulting from the inclusion of otherwise disadvantaged and excluded groups in participation in tourism. The inclusion of these groups in tourism is made possible through financial or other interventions of a well-defined and social nature [24] (p. 29)” shows that social tourism is a civil right as a type of social welfare [11]. The co-word analysis of McCabe and Qiao [2] shows that social tourism is closely related to the keywords of the physical status of tourist (e.g., disabled and health) and age (e.g., senior and children), followed by potential positive consequences of social tourism (e.g., sustainable, quality of life, well-being, and happiness). Social tourism research, in this vein, has attempted to determine what groups have (and do not have) enough accessibility to tourism activity, and why and how (social exclusion and inclusion) [2,8].

Another stream of social tourism research has been conducted with a focus on sustainable tourism and community-based tourism as well [13,15,25]. Destination-focused social tourism research examines the sustainable economic growth of local tourism industries and destinations [2], and the collective well-being of local residents from the public health perspective [26]. Hartwell et al. [26] assert that there is a need for multidisciplinary research collaboration, including destination management, policy, capital, and activities, because excessive tourism activities (i.e., over-tourism) often cause a conflict between the local residents and tourism.

### 2.2. Overview of Growth Mixture Modeling (GMM)

GMM has been developed from the concept of a generalized latent variable modeling framework. Muthén and Muthén [23] proposed the need to integrate person-centered and variable-centered analysis in the context of longitudinal studies; their framework suggests that researchers could simultaneously identify relationships between individuals (e.g., distinct subpopulation—homogenous within categories but heterogeneous across categories)—and between variables (e.g., trends over time, typical structural equation modeling [SEM]) by including the continuous and categorical latent variables. Muthén [27] presented a generalized framework, where traditional SEM is expanded by incorporating categorical latent variables. Similar to the continuous latent variable, the categorical latent variable considers measurement error and multi-item scales. The generalized latent variable modeling framework enables further developments and applications, such as growth curve modeling, multilevel modeling, latent class analysis, missing data modeling, and growth mixture modeling (see Muthén [27] for a further review).

As part of the second generation of SEM [28], GMM can handle measurement errors in the collected data; more importantly, it can be incorporated into a more general latent variable framework by including antecedents and consequences of the changes in the temporal trend. GMM has many advantages over previous analytic methods. Hence, it has been widely adopted in developmental psychology, educational psychology, education, clinical research, and criminology (e.g., Ram and Grimm [22]; Bauer [29]; Harring and Hodis [30]; Jung and Wickrama [31]). For instance, a study on educational psychology investigated heterogeneity in the changes in self-concept in mathematics and its predictive relation to their choice of major [30]. Moreover, in clinical research, Muthén and Muthén [23] revealed the longitudinal classification of heavy drinking measured across ages 18–30 and its relation to alcohol dependence at the age of 30. Thus, GMM allows researchers to identify the trajectories of individual behaviors over time and their connections with the desired target behaviors or status.

GMM has traced its origins back to conventional latent growth curve modeling (LGCM) [23]. LGCM researchers are mainly interested in estimating the average initial status (i.e., intercept in a regression line), the average growth rate (i.e., slope in a regression line), and the variation across individuals’ initial status and growth rate (i.e., variance of intercept and slope) [32]. LGCM also focuses on individual variation in growth trajectory (i.e., intercept and slope) captured by random coefficients. Thus, the continuous latent variables for intercept and slope can vary across individuals to capture individual variability [23]. Furthermore, LGCM can examine individual differences in growth development with respect to the differences in the external situation and individual characteristics [32]. Specifically, it enables researchers to understand individual growth variations by analyzing the relationship of an observed outcome variable to growth pattern and/or to external influence [23]. LGCM application allows researchers to quantify individual differences in their growth and distal outcomes in relation to time-invariant and/or time-variant characteristics (e.g., grade or age).

GMM goes beyond LGCM by considering unobserved heterogeneity in modeling growth differences between samples, while the fundamental assumption of LGCM suggests that all individuals are drawn from a single population [22,23]. In reality, samples are often obtained from multiple populations, regardless of our prior knowledge of group characteristics. An assumption that a single population is unrealistic in many cases may lead to misunderstanding the resulting growth [19]. Everyone may exhibit different characteristics such as personality, educational background, and previous experience, and the differences in individual characteristics may influence the trajectory or the development patterns. This assumption allows different parameter values for mixture components relating to each unobserved subgroup of individuals and captures various trajectory classes based on the differences in growth curve shapes [33]. Substantively, GMM allows researchers to identify multiple unobserved subpopulations to examine the differences in change between unobserved heterogeneity subgroups [22]. Accounting for heterogeneity between samples, GMM is based on a model-based clustering technique called finite mixture modeling to identify latent classes among variables of interest [30]. Guided by theory, GMM empirically estimates the unobserved heterogeneity of the growth trajectory and supports the theoretical argument.

GMM is one of the most generalized forms of the latent variable modeling framework family. It incorporates continuous and categorical latent variables with applications of time-variant and time-invariant predictors and distal outcomes. Other types of latent variable modeling, such as latent class analysis, latent transition analysis, and latent class growth modeling, are special cases of the generalized latent variable modeling [23]. This generalized framework will yield an improved understanding of complex and comprehensive hospitality and tourism phenomena.

In comparison with other methodologies, GMM poses numerous advantages that have been used repeatedly for the same purpose in the hospitality and tourism field. First, GMM takes advantage of all conventional latent modeling techniques (e.g., SEM) like multi-item measurements for the latent variable and the consideration of measurement error. Second, unlike a traditional latent modeling framework, GMM takes the person-centered and variable-centered approaches together. Thus, the model can allow researchers to investigate the changes in the effect over time depending on individual characteristics. Similarly, the third advantage implies that GMM can handle missing data by applying the same methodological treatment with latent variable modeling. Fourth, GMM can be incorporated into a general framework of latent variable modeling. Specifically, the relationships between covariates, class memberships, and distal outcomes are derived from the model specification, rather than post hoc calculation. This simultaneous estimation provides more accurate estimates compared with several separate steps of parameter estimations. Fifth, in comparison with ad hoc segmenting techniques, GMM employs a model-based segmentation approach, which allows the falsifying of suboptimal models. Last, GMM can assume the heterogeneity of samples in the process of estimating the changes in the growth rate. Consequently, GMM helps researchers identify several heterogeneous groups of people who exhibit different levels of growth rate and initial status.

### 2.3. Estimation and Analytic Procedures of GMM

**Figure 1:**
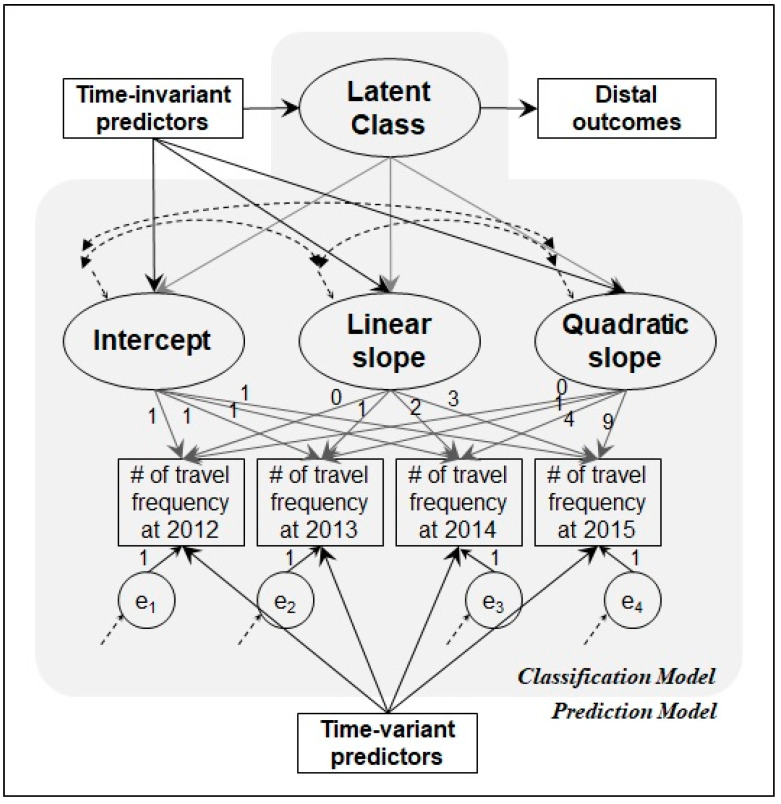
A graphical representation for GMM research. Note(s): Covariance and variance paths are depicted in dotted lines. The numbers on the one-arrow lines are fixed path coefficients.

This section briefly introduces a graphical representation of GMM to provide the underlying analytic background of the current research. Figure 1 depicts a graphical representation of the GMM approach allowing researchers to identify empirically testable hypotheses and brief ideas about modeling strategies. Figure 1 shows that GMM has two major interconnected structural models [34,35], namely, (1) the classification model and (2) the prediction model. In the classification model, there are two notable associations between three components: (1a) observed variables (e.g., travel frequency in each year) are related to continuous latent variables (e.g., intercept, linear slope, and quadratic slope), and (1b) a categorical latent variable (e.g., latent class—potential heterogeneity groups) is estimated from the continuous latent variables. In the prediction model, (2a) travel frequencies regressed on time-variant predictors (e.g., annual income), which help/control model classification. We can identify (2b) who is more likely to belong to a specific latent class by using time-invariant predictors (e.g., gender), and (2c) can also estimate any possible differences in the distal outcomes of respondents (e.g., subjective happiness, quality of life, and/or well-being, which we do not have in our study but would be useful for any follow-up study) by the latent class. The relationships between observed variables, continuous latent variables (trajectory), categorical latent variable (class), time-invariant predictors, and/or time-variant predictors in the diagram can have different parameters in each latent class. The direction of the arrow from the time-invariant predictors to the latent class variable represents the time-invariant characteristics of variables x, namely, socio-economic status or individual enduring personality. Certainly, several properties of the multinomial logistic regression model can be used to explain the relationship between time-invariant predictors and the latent class variable. Moreover, time-invariant predictors and latent class can predict future distal outcomes (change in the outcome), such as academic achievement, subjective happiness, and behavioral intention. The distal outcome can be used to verify the predictive validity of the class memberships based on growth factors, as well as individual characteristics [36]. Lastly, Figure 1 includes a quadratic slope in the model, while only a single linear or non-linear slope variable can also be specified with the changes in the path coefficient for the relationships between latent (i.e., intercept, linear slope, and quadratic slope) and observed variables. Figure 1 also shows that the initial year (i.e., 2012) needs to be set as 0 for both linear and quadratic slopes. The remaining path coefficients for a linear slope latent variable would be fixed at natural numbers (i.e., 1, 2, and 3) starting from 1 to the last number (t − 1), whereas the path coefficients for quadratic slopes would be fixed at the quadratic form of the path coefficient of a linear slope. For the nonlinear model, the first and any other year (usually the second year) are set to be 0 and 1, respectively, while the other years need to be freely estimated.

**Figure 2:**
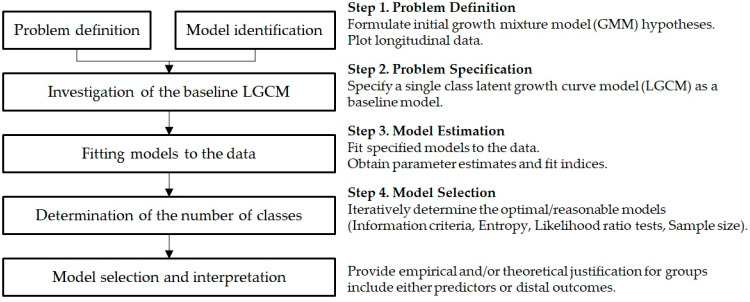
Analytic procedure for GMM research.

This study proposes several procedures based on earlier studies [22,34] as a guideline for using GMM in our field. The application of GMM requires researchers to follow a multistep process for the accurate estimates of parameters and to ensure the validity of the model/results. The suggested procedures for GMM are outlined and explained in Figure 2. A further explanation of the empirical analysis is presented in the latter part of this paper. However, we emphasize the need for guiding theories or reasonable hypotheses regarding trajectories before designing a study using GMM. Although no single theory can guide the heterogeneity of growth factors and longitudinal development process, researchers require highly different theoretical evidence and/or supplementary information to prove the plausibility and interpretability of the results [37]. Specifically, GMM aims to explore the change over time and confirm those identified longitudinal changes in the theory. The initial hypotheses formulated for GMM must be based on guiding theories and substantial evidence, but the actual results would be the foundation for further enrichment of existing theories.

## 3. Research Design

### 3.1. Research Framework

The research framework (see Figure 1) has been developed based on previous studies. At the individual level, travel behaviors may vary from time to time based on circumstances [17,38]. Although the destination choice and subsequent behaviors can change over time in response to changes in circumstance, the general patterns of travel behaviors (e.g., travel frequency) could be related to previous travel patterns, e.g., vacation season, travel frequency, and destination choice. For instance, consistently traveling at a similar time of the year but to another destination(s). In the tourism literature, it is suggested that inertia and traditions regarding vacation throughout the year eventually cause the seasonality of the origin and the destination [39,40]. For example, school holidays, vacations from work, and public holidays substantially impact tourism demand and are generally used for leisure behavior. Therefore, the present study claims that an individual’s travel patterns (i.e., travel frequency in each year) could follow a certain pattern depending on the situation and previous experience. Personal characteristics (socio-demographics, personality, and value) and situation needs/constraints (purpose, party, and travel distance) are inextricably intertwined together, and thus influence travel-related decisions differently [38,41]. Therefore, we assume the heterogeneity of travel patterns and their changes over time in our sample.

### 3.2. Data Preparation

We used the data from the Korea National Tourism Survey for the empirical analysis. The Ministry of Culture, Sports, and Tourism of the Republic of Korea has conducted a recurring longitudinal panel survey of representatives of the South Korean population each year for decades. The survey provides detailed information on travel behaviors and the socio-demographics of Korean households annually. A diary-type survey questionnaire was utilized during data collection to identify the detailed information on each trip that survey respondents took throughout the year, e.g., purpose, destination, length of trip, time of the year, or information sources. Furthermore, this survey collected the data repeatedly from the same respondents over time. These rich and unique characteristics of the data allow for the investigation of the heterogeneity of travel frequency patterns over time. The data can be openly accessed through the Korean Tourism Knowledge and Information System (www.tour.go.kr, accessed on 7 May 2021) [42].

Of the 7588 total survey respondents over a four-year period, the current study only involved those who participated in at least one overnight trip and those who did not miss any survey during the four years. Based on these qualifications, a total of 5054 individual respondents were used for further analysis. This restriction was imperative to the study design because the travel frequency of the first year (i.e., the year 2012 in this study) was the starting point of a trajectory of longitudinal travel frequency. The model cannot be properly estimated without this information. For estimation purposes, all travel-related information (travel frequency, trip purpose, and trip type) were aggregated to the year level from the individual trip level. This transformation allows the incorporation of time-variant and time-invariant variables (demographic characteristics, i.e., age, gender, and income) into the model specification.

### 3.3. Data Analytics

This study follows the analytic procedure described in the previous section (See Figure 2). First, LGCM is employed to identify the overall trajectories of travel frequencies for domestic travel among South Korean tourists. Using four years of longitudinal tourist data, the latent intercept and slopes are estimated from the frequencies of annual domestic travel. This step mainly aims to estimate the overall growth trajectory and the within-subject variation. Second, GMM is employed to explore the heterogeneous subsets of travel trajectory. In this step, a class-specific trajectory for each class is identified independently from the overall level. Third, among several alternative models with different numbers of latent classes tested in the second phase, the optimal model is selected based on the information criteria. The interpretability and the feasibility of the identified solution should also be considered in this process. Lastly, this study would provide empirical justification for the selected model and identify the differences in the characteristics among classes for managerial implications.

## 4. Study Findings

### 4.1. Step 1: Application of LGCM

The first step of the analytic procedure attempted to build a series of models that analyze the trajectories of travel frequencies over time. In doing so, this study first analyzed the descriptive statistics of travel frequencies in our data (see Table 1). The overall trend generally shows an inverted U shape over the four-year period. The average travel frequency in the year 2012 was 2.17. It gradually increased until 2014 (2013: 2.34; 2014: 2.45) and slowly decreased in 2015 (travel frequency: 2.41). This result clearly proves that the overall pattern is not linear but rather quadratic or nonlinear unless it has heterogeneous subgroups. Specifically, this study employed three different LGCMs (i.e., linear, quadratic with centering, and nonlinear models) to choose the best fitting model for the subsequent step. Table 2 provides the model fit indices of three LGCM models. Among them, LGCM02, which is a quadratic model with mean centering, shows the best fit indices (CFI = 1.000, TLI = 1.000, RMSEA = 0.000 [90% CI: 0.000–0.034], and SRMR = 0.002). In addition, we conducted a series of *χ*^2^ difference tests based on the Satorra–Bentler scaled chi-square between the three models. Results showed that LGCM02 is the best model (LGCM01 vs. LGCM02: S-B *χ*^2^ = 85.168, *p* < 0.001; LGCM03 vs. LGCM02: S-B *χ*^2^ = 46.348, *p* < 0.001). Therefore, LGCM02 (i.e., quadratic with mean centering) is the best fitting model for our data. The results indicate statistically significant variations in the intercept, linear, and quadratic slopes of the trajectories in LGCM02 model. This finding implies that the data is not homogeneous with respect to the travel frequency trajectory pattern, thus implying the need for further analysis using GMM.

### 4.2. Step 2: Identification of the Optimal GMM Model

The LCGM results in the first step clearly proving the possibility of several classes based on the growth trajectories for annual travel frequencies over time. To determine the optimal number of classes, the GMM for 1-, 2-, 3-, 4-, 5-, and 6-classes were analyzed. The GMM for more than 6-class was not examined because it does not show any difference in its sample size per class and yields inadmissible solutions for the mean values of intercept, linear, and quadratic variables. Among them, the optimal model was selected based on several criteria proposed by the seminal studies in this area [23,43,44]. The values of Akaike Information Criterion (AIC), Bayesian Information Criterion (BIC), and sample size adjusted BIC (SABIC) for a model with k class should be lower than that for a model with the k-1 class, thus implying that the k class model fits the data better [43,44]. In addition, entropy, which is an indicator representing the quality of the classification, was also used. Entropy ranges from 0 to 1, where the higher entropy value represents the better model [45]. The value of entropy is a standardized measure of classification accuracy based on the posterior probabilities from the corresponding model [46]. Therefore, each sample was assigned to the most likely latent class based on posterior probabilities during the classification. The entropy empirically proves if each individual in the model is classified into the “one and only one category” [37]. However, not only the model fit indices must be considered but also all other empirical evidence, such as the theoretical justification, membership distributions, and the actual pattern of the data [47].

Table 3 indicates the fit indices for six candidate GMM models. Among the six candidates, the 4-class model showed the lowest AIC, BIC, and SABIC values. Moreover, the value of entropy for model 4 was adequate (0.735). In addition to the information criterion values, the sample size per class was also considered to justify the optimal number of classes. Nylund et al. [44] suggest not to use a class with a prevalence of less than 5% due to its low coverage estimates. In reality, the small size of classes could create potential interpretation and generalizability issues. For example, one class in the 5-class model was a relatively smaller size group (2.36%) than the others, and it must be excluded for further empirical analysis. The class membership for the 4-class model was reasonably spread across the classes (8.07%, 14.90%, 32.83%, and 44.20%). Based on this evidence, we selected the 4-class model as the best fitting model that reflects the trajectory of travel frequencies over time. Therefore, this study concluded that the 4-class model had a better fit than the 3- or the 5-class model.

### 4.3. Step 3: Identification of Trajectory

**Figure 3:**
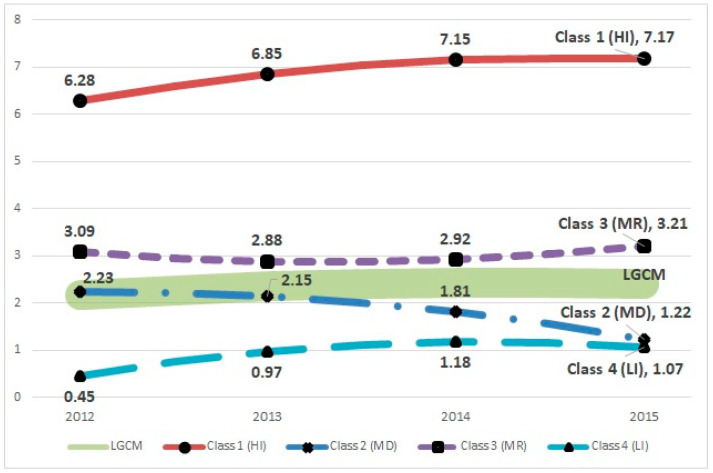
GMM results. Note(s): HI = High-Increasing class; MD = Medium-Decreasing class; MR = Medium-Recovering class; LI = Low-Increasing class; LGCM = Overall latent growth curve model.

Table 4 provides the parameter estimation of latent growth factors based on the best solution for our data, i.e., the 4-class GMM. Our best solution is a quadratic model with mean centering. Thus, the model includes intercept (i.e., initial state), linear, and quadratic terms (i.e., slope). The visual illustration of travel frequency trajectories is presented for an intuitive distinction between the models (see Figure 3). LGCM shows the blurred thick line in the middle, which indicates a weak concave trajectory (*n* = 5054, 100%). By contrast, the 4-class GMM model shows distinctive trajectories across the classes. For instance, class 1, named the ‘high-increasing class [HI]’, shows the highest domestic travel frequency in 2012 (i.e., intercept) and an increasing pattern over time, although the quadratic term is in the negative (class 1: *n* = 408, 8.1%). Classes 2 [medium-decreasing class; MD] and 4 [low-increasing class; LI] illustrate concave trajectories and lower travel frequency than the average in 2015. However, class 4 [LI] starts from almost zero in 2012 (class 2: *n* = 753, 14.9%; class 4: *n* = 2234, 44.2%). While all the three other classes show concave trajectories, class 3 [medium-recovering class; MR] shows a slight convex trajectory above the overall trajectory (class 3: *n* = 1659, 32.8%). The findings of this study confirmed a significant heterogeneity of the travel frequencies that existed among Korean domestic tourists.

### 4.4. Step 4: Understanding of the Characteristics of Each Trajectory Pattern

This study subsequently conducted chi-square and ANOVA tests to investigate and better understand the differences in demographic characteristics between each trajectory. Table 5 illustrates that the four trajectory patterns are independent with regard to the city where they reside in (*χ*^2^ = 95.132, *p* < 0.001), their highest education level (*χ*^2^ = 276.099, *p* < 0.001), and their marital status (*χ*^2^ = 106.622, *p* < 0.001). Furthermore, age (*F* = 44.592, *p* < 0.001) and annual income (*F* = 59.527, *p* < 0.001) are statistically different across four trajectories. In general, the results show that the majority of residents in the HI class (i.e., class 1) live in small- to medium-sized cities, obtain a higher-level education, comprise a relatively higher proportion of the married population, and have the youngest [age] but the highest monthly income. People in MD class (i.e., class 2) reside in big cities, obtain less education, show a slightly lower proportion of married people, are slightly younger, and have above-average income levels. MR class (i.e., Class 3) has similar characteristics to the overall population, i.e., half of them are from big cities, two-thirds obtain lower education level (but are more educated people than the overall population), three-fourths of the population are married, are relatively young, and have higher income level than other groups. The last group, the LI class (i.e., class 4), is more likely to come from a town, shows a relatively high proportion of a lower educated population, has slightly more of a widowed population, and has the oldest age and lowest income in comparison with other groups.

## 5. Discussion and Conclusions

### 5.1. Discussion

The current research explores the heterogeneous latent class with respect to the longitudinal trajectory of annual travel frequency in the Korean domestic travel market so as to examine the sustainable growth of social tourism in Korea. While the overall trend of travel frequency between 2012 and 2015 increased slowly from 2.17 to 2.41, the findings from GMM revealed the four distinct trajectories of travel frequencies among the Korean populations in the meantime. In addition, the findings also note that each group has unique demographic characteristics. Therefore, discovering the four distinctive travel trajectories would help tourism policymakers identify the more- and less-traveling population and thus optimize their available resources (funding, government support, and other subsidies from nonprofit organizations) for the relevant target population.

Although the finding of this study showed that a considerable number of Korean citizens still traveled approximately once per year, the annual travel frequency is not balanced across the classes. One class (HI, class 1) showed overwhelmingly higher figures compared to the frequencies seen in other classes. The HI class covering 8.1% of the population enjoyed the most travel opportunities during the research period. On the other hand, two classes (MD, class 2, and LI, class 4) need more attention from the social tourism perspective. The MD class shows decreasing travel frequency and the LI class still shows the lowest travel frequency over the research period. Compared to the other classes, the members of these two classes received education for a shorter period of time, tend to reside in big cities, are older, and had less income. Furthermore, they have a more complicated marital status structure—with a higher percentage of widowed and divorced.

The findings of the current study demonstrate the inequality of the travel opportunities in the research population, and identifies their demographic characteristics (age, income, and residential area). Therefore, policymakers could utilize this study’s findings as fundamental information for developing an effective tourism policy that promotes public welfare by providing additional travel opportunities and encouraging tourism and leisure activities to those with limited travel experience. Social tourism, as a form of social welfare, could be designed effectively using this study’s findings, thereby enhancing quality of life in general [6,7]. Moreover, this study discovers important insights into public policy decision-making in South Korea by utilizing publicly available national statistical data. Future research could consider including time-variant and time-invariant predictors in the research framework. This step will provide possible solutions for boosting tourism demand at the individual and national levels. Furthermore, the association between domestic and international travel trajectories is a research topic that could provide practical implications for governments to improve national tourism policies.

### 5.2. Potential Application of the GMM Approach in Hospitality and Tourism Research

This study highlights the applicability of the proposed GMM method and the longitudinal study design for future hospitality and tourism research. In general, GMM is applicable to research questions focusing on changes in a certain outcome over time, while interest in the heterogeneity among samples can be added to its utilization. Furthermore, the ability to recognize the heterogeneity within the proposed model would provide more diverse insights and substantial contributions to the fields of hospitality and tourism [20,21]. In this section, we would suggest several possible applications in hospitality and tourism research.

Numerous hospitality and tourism studies have repeatedly confirmed that many people often repurchase the same tourism products or hospitality services over time due to favorable service quality; however, repeated behavior may cause a change in the satiation of affective responses [48,49]. This type of research problem can be solved by using GMM in a longitudinal manner. In this case, the level of satisfaction (or revisit intention) for each repurchase experience is the main interest variable. Applying GMM to this case allows us to capture the pattern of satiation over time, the heterogeneity within the sample, and potential determinants or outcomes.

GMM can also be useful for data at the firm level because the performance of hospitality firms is often dynamic in nature depending on the seasonality and/or economic situation [50,51]. The desired outcomes (or variables of interest) can be the annual performance of the hospitality company, the number of service failure incidents in each year, and any periodical information that describes the firms’ performance. Other possible examples of longitudinal analysis in hospitality and tourism fields would be identifying how the hospitality firms’ revenue eventually changes after launching a new advertisement, the relationship between consumers’ levels of satisfaction and repeated purchases, how emotional labor employees manage stress after completing an emotional intelligence training program, or how tourists use social media differently before, during, and after travel [52,53]. By employing the GMM approach to the longitudinal data, researchers can identify the dominant patterns of changes in the variable of interest among samples and the possible influencing factors from the research findings. For example, restaurant owners or policymakers could identify the underlying patterns of food risk (e.g., food poisoning incidents) in the city and subsequently identify the potential area that needs special care or the potential policy that needs to be revised.

Lastly, GMM is widely adopted by developmental psychologists. In reality, hospitality companies have put effort into their employees’ education and focused on retaining successfully educated employees [54,55]. Similarly, a hospitality company can use GMM to determine the influencing factors during the training/education programs and identify possible solutions for the previous question, which require an improved understanding of the development pattern and the characteristics of employees in each trajectory. In this example, the variable of interest would be the level of expertise, the number of the task completed, and job satisfaction. The findings from these studies would help human resource managers improve their understanding of their employees and design an effective education and training program for them.

### 5.3. Challenges in GMM for Hospitality and Tourism Research

While GMM evidently possesses a great potential to contribute theoretically and practically to hospitality and tourism research, future studies should incorporate several important considerations to actually implement the GMM approach in the framework. First, GMM, as a longitudinal research method per se, requires data collection from the participants at multiple times (e.g., more than three times due to the model identification condition) without any intervention or with very limited intervention. GMM often requires a heavy computational analytic process especially as the number of classes increases, which recommends a large sample size for practical application [22]. Therefore, researchers must exert substantial time, effort, and money to simply obtain the primary data for their research. However, sample attrition problem often arises during the data collection process. Possible solutions include collaboration between hospitality companies, use of publicly available data, or survey of panel members over time [18]. Second, while using the GMM approach, researchers often fall into a trap, i.e., focusing on the empirical evidence rather than the theory itself. As part of the SEM family, the research framework/model should be based on theory and theoretical discussion (i.e., a confirmatory approach), rather than the exploration of the possible outcome from the data (i.e., an exploratory approach) [23,37,43]. Regardless of these challenges for the method application, GMM could address many important research questions in our field via an advanced and rigorous research design. Researchers should revisit their own research questions again using GMM to address unanswered or inaccurately answered questions.

### 5.4. General Conclusion

This study explored and identified certain subpopulation clusters with unequal travel experiences from the social tourism perspective. To this end, the study applied the GMM technique with four-year-long longitudinal travel frequency data in the Korean domestic travel market. The study found four distinctive longitudinal clusters concerning the annual travel frequency. Policymakers are expected to make the best use of the current study findings when working on policies regarding social tourism. The present paper also benefits academic researchers by providing practical guidelines on how to apply GMM. GMM allows researchers to describe changes in the key outcome variables over time and subsequently cluster samples into several groups simultaneously depending on the trajectories. This advantage could provide fruitful insights and discussions for academia and practitioners in our field.

## Figures and Tables

**Table 1:** Descriptive statistics of annual domestic travel figures.

Year	Mean	S.D.	Min.	Max.	Skewness	Kurtosis
2012	2.17	2.50	0 (26.4%)	26	2.52	11.44
2013	2.34	2.79	0 (25.6%)	39	3.08	18.84
2014	2.45	2.62	0 (20.3%)	26	2.64	12.04
2015	2.41	2.81	0 (21.4%)	39	3.60	26.07

**Table 2:** LGCM models.

Model	*χ* ^2^_(*df*)_	RMSEA (90% CI)	CFI	TLI	SRMR		Variance	
						Intercept	Slope	Quadratic
LGCM01 Linear	96.721_(*5*)_ ***	0.060 (0.050–0.071)	0.956	0.947	0.035	5.019 ***	0.433 ***	
LGCM02 Quadratic ^a^	0.607_(*1*)_	0.000 (0.000–0.034)	1.000	1.000	0.002	5.046 ***	0.562 ***	0.217 ***
LGCM03 Non-linear	56.909_(*3*)_ ***	0.060 (0.047–0.074)	0.974	0.948	0.036	9.457 *	4.697	

Note(s): RMSEA = Root mean square error of approximation; CI = Confidence interval; CFI = Comparative fit index; TLI = Tucker-Lewis index; SRMR = Standardized root mean square residual. ^a^ The Quadratic model was estimated with centering. *** *p* < 0.001, ** *p* < 0.01, * *p* < 0.05.

**Table 3:** Fit indices for GMM models.

Model	H0 LL_(*p*)_	AIC	BIC	SABIC	ALMR LRT	BLRT	Entropy	Sample Size per Class ^a^					
								1	2	3	4	5	6
1-Class	−44,460.88_(*13*)_	88,947.76	89,032.63	88,991.32	-	-	-	5504 (100.0)					
2-Class	−41,186.91_(*20*)_	82,413.82	82,544.38	82,480.82	6654.66 ***	6732.91 ***	0.781	1026 (20.30)	4028 (79.70)				
3-Class	−40,348.61_(*24*)_	80,745.22	80,901.89	80,825.63	3827.60 ***	3872.49 ***	0.725	519 (10.27)	2050 (40.56)	2485 (49.47)			
4-Class ^★^	−40,196.86_(*28*)_	80,449.72	80,632.50	80,543.53	3025.43 ***	3060.90 ***	0.735	408 (8.07)	753 (14.90)	1659 (32.83)	2234 (44.20)		
5-Class	−41,123.21_(*28*)_	82,302.42	82,485.20	82,396.22	397.65 *	409.29 ***	0.786	2892 (57.22)	1148 (22.72)	119 (2.36)	351 (6.95)	544 (10.76)	
6-Class	−41,071.19_(*31*)_	82,204.37	82,406.74	82,308.23	334.75 ***	344.57	0.824	402 (7.95)	869 (17.19)	2941 (58.19)	412 (8.15)	319 (6.31)	111 (2.20)

Note(s): LL = Log-likelihood; AIC = Akaike information criterion; BIC = Bayesian information criterion; SABIC = Sample size adjusted BIC; ALMR LRT = Adjusted Vuong-Lo-Mendell-Rubin likelihood ratio test; BLRT = Bootstrap likelihood ratio test. ★ indicates the optimal solution based on the model fit indices. ^a^ The number in parenthesis indicates the percentage of the corresponding class. *** *p* < 0.001, ** *p* < 0.01, * *p* < 0.05.

**Table 4:** Parameter estimation of latent growth factors for the 4-class GMM.

Class	Trajectory Variable	Mean	Variance
Class 1 (High-Increasing class)	Intercept	7.032 ***	19.912 ***
	Linear	0.297 ***	5.134 ***
	Quadratic	−0.137	3.543 ***
Class 2 (Medium-Decreasing class)	Intercept	2.015 ***	0.080 *
	Linear	−0.337 ***	0.000 ^a^
	Quadratic	−0.129 **	0.000 ^a^
Class 3 (Medium-Recovering class)	Intercept	2.873 ***	1.210 ***
	Linear	0.039	0.722 ***
	Quadratic	0.123 ***	0.000 ^a^
Class 4 (Low-Increasing class)	Intercept	1.111 ***	0.000 ^a^
	Linear	0.207 ***	0.000 ^a^
	Quadratic	−0.156 ***	0.000 ^a^

Note(s): ^a^ Due to the inadmissible solution, the corresponding variance was fixed at zero in the model, assuming no variance of the corresponding factor. *** *p* < 0.001, ** *p* < 0.01, * *p* < 0.05.

**Table 5:** Differences in demographic characteristics between trajectories.

Demographic	Class 1 (HI)	Class 2 (MD)	Class 3 (MR)	Class 4 (LI)	Total	χ^2^_(*df*)_/F_(*df*)_
City Size (%)						95.132_(*6*)_ ***
Big city	35.5	48.5	44.5	46.6	45.3	
Small/Middle city	46.1	29.9	38.0	27.9	33.0	
Town	18.4	21.6	17.5	25.5	21.7	
Education (%)						276.099_(*21*)_ ***
No education	1.7	2.7	1.6	5.0	3.3	
Elementary school	3.4	11.0	6.9	16.6	11.5	
Junior high school	6.1	11.6	7.8	12.1	10.1	
High school	32.4	38.5	36.2	34.0	35.3	
2-year college	15.2	9.6	13.0	7.7	10.3	
4-year university	36.0	24.8	29.6	22.6	26.3	
Graduate (Master)	4.2	1.7	3.6	1.7	2.5	
Graduate (Doctor)	1.0	0.1	1.2	0.3	0.6	
Marriage (%)						106.622_(*9*)_ ***
Single	13.7	24.0	19.3	23.2	21.3	
Married	83.1	68.3	75.3	65.4	70.5	
Widowed	2.5	6.4	3.6	9.0	6.3	
Divorced	0.7	1.3	1.7	2.3	1.9	
Age						44.592_(*3*)_ ***
Mean	43.57 ^(a)^	46.72 ^(b)^	44.32 ^(a)^	50.34 ^(c)^	47.28	
Std. dev.	(13.99)	(18.18)	(15.63)	(19.22)	(17.80)	
Annual Income (1000 KRW)						59.527_(*3*)_ ***
Mean	49,920 ^(c)^	39,247 ^(b)^	47,405 ^(c)^	35,975 ^(a)^	41,340	
Std. dev.	(26,782)	(29,420)	(37,032)	(23,850)	(30,321)	

Note(s): HI = High-Increasing class; MD = Medium-Decreasing class; MR = Medium-Recovering class; and LI = Low-Increasing class. ^a^ Duncan Post Hoc Test was performed for Age and Annual Income. Distinct subgroups were labeled with a superscription of ^(a)^, ^(b)^, and ^(c)^. *** *p* < 0.001, ** *p* < 0.01, * *p* < 0.05.

## Data Availability

Publicly available datasets were analyzed in this study. This data can be found here: [https://www.tour.go.kr, accessed on 7 May 2021].
